# The proposed rule of the FDA banning menthol as a characterizing flavor

**DOI:** 10.18332/tid/149993

**Published:** 2022-05-27

**Authors:** Kelsey Romeo-Stuppy

**Affiliations:** 1 Action on Smoking and Health, Washington, United States

**Keywords:** menthol, ban, litigation, flavor, legislation

On April 28, the United States Food and Drug Administration announced a draft rule banning menthol^1^. This is an exciting step forward for public health in the United States, but this rule has been a long time coming.

With the Family Smoking Prevention and Tobacco Control Act of 2009^2^, Congress took an important step to prevent youth smoking by banning flavored cigarettes; however, with lobbying from the industry, they made an exception for menthol. In 2013, a citizen’s petition was submitted to the FDA by nineteen public health organizations, urging the FDA to remove menthol cigarettes from the market^3^. Although required by law, the FDA failed to respond to the petition.

After over seven years of waiting, a lawsuit was filed by the African American Tobacco Control Leadership Council (AATCLC), Action on Smoking and Health (ASH), the American Medical Association (AMA), and the National Medical Association (NMA) in order to force the FDA to respond to the petition. The plaintiffs are represented by Pollock Cohen LLP. The complaint^4^, initially filed on 17 June 2020, asserts that contrary to the duties imposed by the Family Smoking Prevention and Tobacco Control Act (‘Tobacco Control Act’), the FDA has failed to act on menthol cigarettes. The lawsuit prompted a much overdue response from the FDA to the petition. On 29 April 2021, the FDA announced that they would promulgate a rule to ban menthol cigarettes^5^, and on 28 April 2022, they issued the first draft of that rule^1^.

This announcement is a draft text of the actual rule and is the first time advocates have been able to see the text of the rule. Now, civil society and others, including the tobacco industry, will have an opportunity to give comments for 60 days. After the comment period closes in July, the FDA will review all of the comments and put together a final draft of the rule. The final rule will go into effect one year after it has been published.

Unfortunately, this is not a terribly fast timeline and lives are at stake. Action on Smoking and Health (ASH) and partners plan to keep international pressure on the FDA by utilizing human rights mechanisms. In the United States, the tobacco industry has heavily targeted the African American community with advertising for menthol cigarettes, and as a result, 9 out of 10 African American people who smoke, smoke menthols. This makes menthol a health equity and social justice issue as well as a tobacco control issue. The United States is due for review before the Committee on the Elimination of Racial Discrimination (CERD), the body of independent experts that monitors the Convention on the Elimination of All Forms of Racial Discrimination, a human rights treaty to which the United States is a party. ASH and partners plan to ask the committee (through both a written and oral statement) to urge the United States to move as quickly as possible on the menthol ban in order to save lives.

In addition to keeping up international pressure, we encourage local and state advocates to continue to push for menthol bans in their jurisdictions. While the draft rule is an exciting step, we are not finished with the fight yet. We must keep pressure on the FDA until all Americans are protected from the harms of menthol.

## Data Availability

Data sharing is not applicable to this article as no new data were created.
